# Remarks on Tying the Aorta

**Published:** 1818-03

**Authors:** Samuel Young


*For the London Medical and Physical Journal.*

*Remarks on tying the Aorta.* By SAMUEL YOUNG, Esq.

I HAVE this moment the recently published Essays of Messrs. Cooper and Travers before me; and, in reading the case where the aorta was tied by Mr. Cooper, so remarkable a deficiency of physiological foresight and deduction appears on the face of it, that I turn for a short time from the course of other pursuits to draw the attention of your readers to it. And I do this the more promptly, because I have ever felt most forcibly the justice of the impression, that the errors of eminent men are proportionably mischievous according to their eminence and influence. Measuring, therefore, oversight and false deduction according to this rule of eminence and celebrity, and surrounded as the author is by pupils as a public teacher, the importance that any error from such a source should not pass current, at least without notice, is in itself so evident, that to enlarge further on this point would be superfluous.

Of the dexterity and delicacy exhibited, if such latter term may be applied to an operation where the human aorta was tied, one can only speak in terms of admiration, as they appear most prominent in the description of the case as far as the operation went; and in the *proof* also, as exhibited in the examination after death, where it was made evident that *nothing* but *aorta* was included in the ligature.

Of the necessity of the operation, from the deficiency and lost state of the vessel in the aneurismal sac, and from all the circumstances of the case, it is also evident that passing the ligature was the only thing left, among the *last snatches* of chance, to save or prolong the life of the patient.

But the point to which I wish to draw the attention of your readers is, the apparent want of physiological deduction before, as well as pathological research after, the death of the patient; as appears in the report now made public, as well also as the author's own practical conclusions after the ligature was passed, and the symptoms which followed.

As far as the examination went after the death of the patient, nothing could be more satisfactory. The abdomen and its contents were free from inflammation or any unnatural colour, and large coagula plugged the aorta itself, as well as the iliacs. "All were gratified to see the artery so completely shut in forty hours." But here the striking deficiency in the report of the case is, that the examination should have ended here. After such an operation, and such symptoms following as they did, one cannot but be struck with surprise that no report follows about the contents of the chest, of the state of the vessels of the heart and lungs; and especially when all is silence about the state of the brain itself, and the condition of the blood-vessels,—whether they were empty or turgid, or whether any extravasation had taken place or not.

All such enquiry and examination seems to have been passed by, though the aorta itself had been tied, and though, during the man's life, after the ligature was made, he complained, as seen by the notes of the case, "of pain all over his body, more particularly in his head, and the carotids beating with considerable force;" and though, as seen by another part of a note, when in a sinking state, "he appeared to have an uneasiness about the heart, as he kept his hand upon the left breast."

The report, therefore, so far as these points are involved with the nature of the operation, and the symptoms noted after the ligature was made, is evidently deficient in physiological anticipation, as well as in pathological research.

Deficient in physiological anticipation, because the consequences, after the aorta was tied, of retrograding the whole circulation of blood back into the vessels of the head and chest, except such portion as would escape by collateral vessels, to circulate through the lower extremities, ought naturally to have been anticipated and provided against; so far, at least, as relieving the evidently overcharged state of the vessels of the head by blood-letting, by opening one or both of the jugulars, and thereby relieving also the evident congestion about the vessels of the heart.

And deficient in pathological research (after death), when so evidently directed by previous symptoms, by the violent beatings of the carotids, by the pain and heat of the head, the anxiety of countenance, by the uneasiness expressed about the heart, &c. in the absence and oversight of all examinations whatever into the state of the contents of the head or chest. Parts which, by *a priori* physiological conclusion, must have been expected to have been materially affected by such an operation as a ligature on the aorta; and the vessels of which, as seen by the evidence of symptoms after the operation, were so evidently disturbed and surcharged.

We now come at once to the author's conclusions as to the cause of death in this case; and which appear to be entirely opposed to the facts of the case. And the consideration of the cause of death in this instance, is the more important, since a most serious practical question becomes involved; as it is probable that placing a ligature on the aorta will now rank among practical operations, as the author himself seems to have made up his mind on the subject, when he observes, "In an aneurism, therefore, similarly situated, the ligature must be applied before the swelling has acquired any very considerable magnitude."

There appears to have been a difference in the degrees of heat between the two extremities; the aneurismal limb being seven degrees lower than the other. And, to this cause, the author attributes the death of the patient.—"His death appears to me to been owing to want of circulation in the aneurismal limb."—These are his words.

This would appear a most deficient cause, indeed, for death in this case; because, although the limb was cold, and had a livid appearance, no gangrene took place; and because, we know also, that, where the artery of the thigh has been high tied up, equal coldness and lividness of appearance have existed, and where the limb has ultimately recovered its natural temperature.

Indeed, the author here is too inconclusive to be rightly intelligible.

On the evidence of this case, therefore, I feel entirely at issue with the author in assigning the cause of death. Instead of the impaired state of circulation through the aneurismal limb being the cause, I should say, the cause of death arose from the congested state of the blood-vessels of the head, heart, and lungs, &c. in consequence of the ligature upon the aorta, as was evinced by the beatings of the carotids, by the heat and pains of the head, the anxiety of countenance, by the uneasiness expressed about the region of the heart, and by the general pains felt about all the upper part of the body. In short, generally speaking, these symptoms would have existed, and the same result have taken place, if the ligature had been so passed, whether an inguinal aneurism had existed or not.

The analogy betwixt the tying up of the aorta of a dog and that of a man, if put at all in parallel, would appear very much over-strained. That which appears, and may be proved, very practicable in the dog, only makes it a barely abstract possibility in the instance of the man. There can be no sort of practical comparison between the vascular systems of the two, and the parts that must necessarily be affected, when such a violence is committed, as tying up such a vessel as the aorta. What practical parallel can possibly be made, for example, between the brain of a dog and that of man, when labouring under vascular congestion? The comparison is just the leg of a fly to that of a dog; and, because the one seems to bear the loss of a limb or two with impunity, it might equally be argued, that the other might as equally sustain the same abridgment. Such forced analogies beget a love of operation beyond the limits of common sense. And I say thus much, not that the author should be less esteemed, but that truth and real practical facts may be more respected. And that students should feel, that they have other duties to discharge as surgeons, as well as running about the country performing extraordinary operations, and tying up of aortas.

39, *Lower Brook-street;*
*Feb.* 14, 1818.

